# Application of AI to Ultrasonographic Images to Aid the Clinical Care of Pregnant Women With Pre-eclampsia in Uganda: A Protocol for a Pilot Study

**DOI:** 10.7759/cureus.101406

**Published:** 2026-01-12

**Authors:** Mugyenyi R Godfrey, Fredrick Atwiine, Esther C Atukunda, Leo Anthony Celi, Rogers Mwavu, Fred Kaggwa, Jessica E Haberer, William Wasswa

**Affiliations:** 1 Obstetrics and Gynaecology, Mbarara University of Science and Technology (MUST), Mbarara, UGA; 2 Obstetrics and Gynaecology, Divine Mercy Hospital-Father Bash Foundation, Mbarara, UGA; 3 Mbarara University Data Science Research Hub, Mbarara University of Science and Technology, Mbarara, UGA; 4 Pharmacy, Mbarara University of Science and Technology, Mbarara, UGA; 5 Medicine, Beth Israel Deaconess Medical Center, Harvard Medical School, Boston, USA; 6 Computing and Informatics, Mbarara University of Science and Technology, Mbarara, UGA; 7 Internal Medicine, Harvard Medical School, Massachusetts General Hospital, Boston, USA; 8 Computing and Informatics, Mbarara University Data Science Research Hub, Mbarara University of Science and Technology, Mbarara, UGA; 9 App Development, Global Auto Systems LTD, Mbarara, UGA

**Keywords:** artificial intelligence(ai), clinical data science, machine learning in medicine, pre-eclampsia, ultrasonographic images

## Abstract

Background

Artificial intelligence (AI) refers to computer systems designed to perform tasks requiring human intelligence, including medical diagnosis. AI methods have enhanced diagnostic processes across various diseases. In obstetrics, conditions such as pre-eclampsia are typically assessed using USG, yet access to these services and trained sonographers remains limited. Automated diagnosis using AI applied to stored images offers an opportunity to improve maternal and fetal outcomes. In Uganda, progress in integrating AI into obstetric care has been minimal, despite the high burden of complications. This study aims to create a Doppler USG image database, annotated for machine learning models to predict pre-eclampsia complications.

Methods

This cross-sectional study will enroll 150 pregnant women seeking obstetric USG services at Divine Mercy Hospital, Mbarara City, Uganda. Participants will be recruited consecutively from the outpatient department, with half expected to have pre-eclampsia. Sociodemographic, obstetric, and clinical data will be systematically collected, de-identified, and linked to corresponding Doppler ultrasonographic images. Data elements will include acquisition parameters, key Doppler indices, and anonymized demographic information. All data will be securely stored in a structured repository hosted by the Data Management and Analysis Core (DMAC) of the Mbarara University Data Science Research Hub. Images will be annotated using a standardized protocol by trained experts and linked with structured clinical metadata. The dataset will be partitioned into training, validation, and test subsets. Machine learning approaches will include convolutional neural networks, ensemble learning, and transfer learning. Performance will be evaluated using cross-validation, area under the receiver operating characteristic (ROC) curve (AUC), precision, recall, and F1-score, with hyperparameter optimization via grid and Bayesian search.

Results

The primary outcome will be a de-identified, annotated dataset of obstetric Doppler ultrasonographic images linked to structured clinical metadata, including the amniotic fluid index, fetal heart rate, umbilical and cerebral arteries, uterine arteries, and placenta. Images will undergo standardized preprocessing and dual expert review, with discrepancies adjudicated by a third reviewer. The secondary outcome is a multimodal AI tool predicting maternal and fetal complications of pre-eclampsia. Data collection targets 150 participants, funded by the National Institutes of Health (NIH) Data Science Initiative for Africa.

Discussion

In settings with limited access to expert imaging, this study will develop a high-quality annotated dataset and a context-specific machine learning model trained on images from pre-eclamptic and non-pre-eclamptic pregnancies. It will be among the first obstetric AI datasets derived entirely from an African population, addressing global gaps in representation. Aligned with Uganda’s Digital Health Strategy, the study supports innovative tools to strengthen clinical decision-making, improve maternal and perinatal outcomes, and advance AI integration into routine obstetric care in low-resource settings.

Conclusion

This study will generate one of the first annotated obstetric Doppler ultrasound datasets from sub-Saharan Africa. The findings will inform potential integration of AI tools into routine maternal healthcare.

## Introduction

Obstetric ultrasonographic imaging, also known as prenatal or pregnancy ultrasound scans (USS), plays a vital role in obstetric care by providing a safe, non-invasive, and highly effective method for monitoring fetal development and maternal health throughout pregnancy [1, 2]. It is used for confirming pregnancy, estimating gestational age, detecting multiple pregnancies, and assessing fetal growth, anatomy, and well-being [3]. USS is also instrumental in identifying congenital anomalies, placental abnormalities, biological sex, and pregnancy or fetal complications, although most guidelines still recommend clinical assessment as a cost-effective screening tool to identify growth restriction [4, 5]. Doppler ultrasound further aids in evaluating blood flow to detect conditions like fetal growth restriction and preeclampsia [6-8]. Regular ultrasound assessments enhance prenatal care by facilitating early diagnosis, guiding interventions, and improving pregnancy outcomes [9].

Advancements in medical technology have further enhanced obstetric care, with artificial intelligence (AI) emerging as a powerful tool in disease diagnosis, including the detection and management of diseases, a process traditionally performed by humans. AI refers to computer systems that perform tasks that typically require human intelligence, mimic human behavior, and can be programmed to complete automated processes [10], including medical diagnosis. AI-driven technologies are revolutionizing disease diagnosis and management by improving the accuracy and efficiency of medical assessments. Machine learning, a branch of AI, focuses on creating algorithms and statistical models that allow computers to learn from data and make predictions or decisions with minimal human input [11]. Machine learning algorithms can analyze vast amounts of ultrasound imaging data to detect anomalies such as fetal growth restriction, congenital abnormalities, and placental disorders with greater precision. Ultrasonographic images have previously been used in high-income countries to develop diagnostic predictive models for pediatric appendicitis [12] and determination of fetal sex [13]. AI-driven technologies are revolutionizing disease diagnosis and management by improving accuracy and efficiency of medical assessments. A recent SWOT-based review of AI progress in clinical medicine reported that AI has been associated with improved diagnostic accuracy and operational efficiency across clinical applications [14]. In low- and middle-income countries, information on the use of USG images and machine learning in diagnosing fetal and maternal complications for early treatment is not available.

Although training clinicians to perform USS in their routine clinical practice is more cost-effective in low-resource countries, because clinicians are able to correlate USG and clinical situations better to guide patient management, this would provide a good solution for the scarcity of sonographers in these settings. Currently, clinicians request USS examinations, which sonographers perform, providing written reports and images for clinical decision-making. This process is not only time-consuming, but often there is a scarcity of qualified radiographers or sonographers to perform the examinations and limited expertise to interpret the images. Using AI-powered decision-support systems, obstetric disease such as pre-eclampsia and its complications could be diagnosed in time for appropriate management [15]. In the 2022/2023 financial year, 75% of pregnant women in Uganda received antenatal care from midwives and delivered at lower-level health facilities that lack radiologists and sonographers. These facilities largely rely on diploma-level midwives with minimal sonographic training, limiting their ability to diagnose complex cases [16]. Consequently, many women cannot access essential USG services, resulting in undetected complications, misdiagnoses, and delayed treatment. AI offers promising solutions to improve clinical diagnosis and address these obstetric challenges. In Uganda, a study has shown that AI can provide quality and objective decision support in screening for cervical cancer [17]. AI-powered tools could enable lower-level clinicians to achieve accurate diagnoses. However, there is currently little information on efforts to integrate AI into routine care for pregnant women in Uganda, yet the burden of obstetric complications such as those associated with pre-eclampsia is high and is greatly associated with maternal and perinatal deaths. The primary objective for this pilot project is to create a de-identified, annotated Doppler USG dataset comprising at least 900 images from 150 pregnant women (75 with pre-eclampsia, 75 without) at Divine Mercy Hospital-Father Bash Foundation, Uganda. The secondary objective is to develop and evaluate a multimodal AI tool capable of predicting maternal and fetal complications of pre-eclampsia with target performance metrics. We hypothesize that a machine learning model trained on locally sourced Doppler USG images combined with clinical metadata can predict pre-eclampsia complications with clinically acceptable accuracy.

## Materials and methods

Study design

This will be a cross-sectional study of pregnant women seeking obstetric USG services at Divine Mercy Hospital, Mbarara City, Uganda.

Study setting

Divine Mercy Hospital-Father Bash Foundation is a private for-profit hospital located in Kamukuzi Division, Mbarara City, about 270 km southwest of Kampala, the capital city of Uganda. It offers both general and specialized medical services [18] and serves a population from about 15 districts surrounding Mbarara, but receives patients from wider western Uganda and beyond. Divine Mercy Hospital has a bed capacity of 100 and receives, on average, 16,805 patients (9,990 females and 6,815 males), with 35,000 outpatient visits annually. The Radiology Department of Divine Mercy Hospital performs approximately 5,000 obstetric ultrasound examinations annually. The department employs two radiographers supported by a radiologist, who provide a written report for every examination performed.

Study population

We shall enroll 150 pregnant women attending antenatal care and seeking an obstetric USG examination at Divine Mercy Hospital, half of whom will have pre-eclampsia. Pre-eclampsia will be evidenced by the presence of high blood pressure >140/90 mmHg and proteinuria of 2+ and above after 20 weeks of gestation, and participants must be willing to consent. Severely ill patients and those in need of immediate delivery will be excluded.

Sample size determination

This study will collect ultrasonographic images from pregnant women attending antenatal care at Divine Mercy Hospital. Our target is to collect an equal number of images from all participants, which will be annotated for algorithm model development to predict maternal and fetal complications for early treatment and delivery. According to hospital records, about 75 pregnant women out of about 5,000 managed at Divine Mercy Hospital annually have pre-eclampsia. For this pilot study, we propose to recruit approximately 150 pregnant women (75 with pre-eclampsia and 75 without). From each participant, we will collect 6 images of the fetus and the placenta, giving an overall total of 900 ultrasonographic images.

Sampling procedure

Participants will be recruited from the outpatient department of Divine Mercy Hospital using a purposive sampling method for women with pre-eclampsia and a consecutive sampling method for patients without pre-eclampsia. On each data collection day, all women requested to have a sonographic examination will be approached for informed consent for participation in this study before recruitment.

Study outcomes

The primary outcome will be an annotated dataset of quality Doppler ultrasonographic images. The secondary outcome will be an automated AI tool to predict maternal and fetal complications of pre-eclampsia for early treatment or timing of delivery.

Data collection procedures

Data will be collected using a customized smart mobile application, SonoAI. This SonoAI mobile application will be developed by a dedicated research team of data scientists, information technology experts, and clinicians (obstetricians/gynaecologists, radiologists, and ultrasonographers). The SonoAI mobile application shall include a user-friendly interface for data entry and participant enrollment, as well as a data-driven dashboard for image upload and data management, including quality assurance, data review, and labelling.

This SonoAI mobile application will be used to collect the following data: (a) Sociodemographic data (including age, weight, height, BMI, occupation, marital status, and tribe). Each participant shall be assigned a unique participant identifier. (b) Obstetric characteristics (last normal menstrual period, gestational age, gravidity/parity, previous pregnancy complications, previous mode of delivery, and history of hypertension). (c) Diagnosis and laboratory data (clinical diagnosis, ultrasound findings, booking blood pressure, blood pressure at enrollment, and other features of severe pre-eclampsia). The laboratory data will include basic haematological tests routinely done in patients with pre-eclampsia, such as CBC, platelet count, and urine protein.

The SonoAI dashboard, a data analytics platform, will be used to register users, upload images, archive data, and support data management processes. The dashboard will mainly be used for: (a) User management, including creating user login accounts, managing access logins, and tracking data entered by each user. (b) Uploading images into participant folders. (c) Data quality control (data review in both longitudinal and report formats, data download, data editing, and deletion). (d) Data labelling (assigning images to the labellers and managing the data labelling process, review, and approvals). (e) Linking and archiving images and metadata to the Data Management and Analysis Core (DMAC) storage infrastructure of the Mbarara University Data Science Research Hub.

Participant recruitment and consent

**Figure 1:**
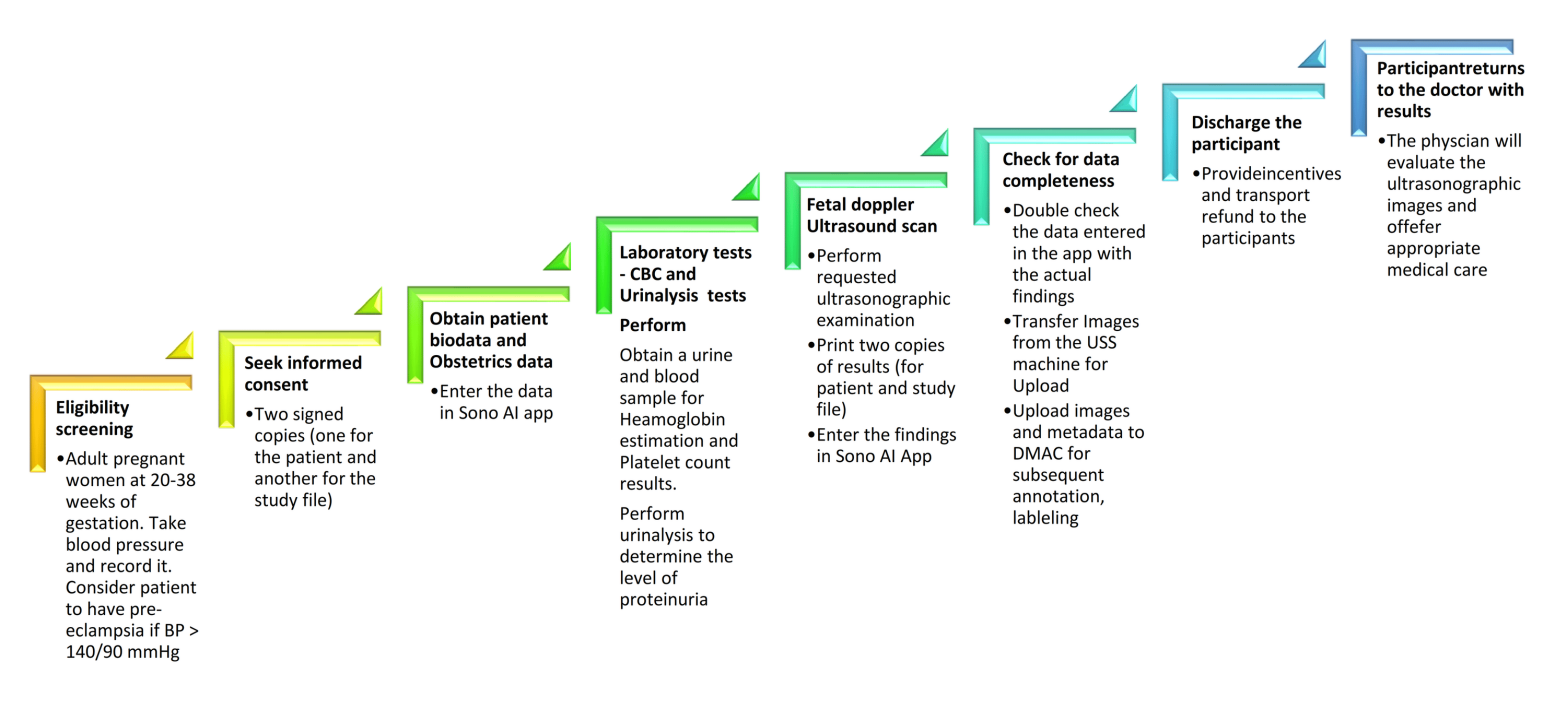
Study procedures and standard operating procedures for participants at enrollment.

Adult pregnant women between 20 and 38 weeks of pregnancy will be recruited from the outpatient department of Divine Mercy Hospital using consecutive sampling for women with normal blood pressure and purposive sampling for women with pre-eclampsia. This will occur after they have been seen by a clinician who would have requested an obstetric ultrasonographic examination. Participants will undergo eligibility screening, and consent will be obtained from those eligible. Sociodemographic data shall be obtained and entered directly into the SonoAI application. Laboratory and ultrasonographic examinations will be performed following standard operating procedures outlined in Figure 1 and Table 1 below. All pregnant women with a request for ultrasonographic examination between 20 and 38 weeks of gestation will be approached by a trained research assistant for consent before recruitment. Information on the study will be provided in a language clearly understood by the participant. Written consent shall be obtained. A qualified sonographer or radiologist shall, at the time of examination, further explain the imaging procedure, including answering any questions before the examination. In addition, a chaperone shall be present during all ultrasonographic examinations.

**Table 1:** Standard operating procedures for obtaining Doppler ultrasonographic images using the Edan Acclarix LX25 diagnostic ultrasound system (version 1.5). SOP: Standard Operating Procedure(s); AFI: Amniotic Fluid Index; MCA: Middle Cerebral Artery; UA: Umbilical Artery; UtA: Uterine Artery; PW: Pulsed-Wave (Doppler); PSV: Peak Systolic Velocity; S/D ratio: Systolic-to-Diastolic ratio; PI: Pulsatility Index; RI: Resistance Index; MoM: Multiples of the Median; FHR: Fetal Heart Rate; cm: centimeter(s); bpm: beats per minute; 2D/3D/4D: Two-dimensional/Three-dimensional/Four-dimensional; DICOM: Digital Imaging and Communications in Medicine; CLAHE: Contrast Limited Adaptive Histogram Equalization; ROC: Receiver Operating Characteristic; AI: Artificial Intelligence; CNN: Convolutional Neural Network; SGD: Stochastic Gradient Descent; DMAC: Data Management and Analysis Core; REC: Research Ethics Committee; ROI: Region of Interest.

SOP / Examination	Purpose	Patient preparation	Image acquisition	Measurement / Calculation	Documentation / Notes
Amniotic Fluid Index (AFI)	Measure amniotic fluid volume and assess fetal well-being	Supine with slight left lateral tilt; expose abdomen (pubic symphysis to fundus)	Grayscale (B-mode). Divide uterus into 4 quadrants using umbilicus as landmark (vertical linea alba + horizontal line through umbilicus). In each quadrant, identify deepest vertical fluid pocket free of cord/fetal parts; measure pocket depth (cm) perpendicular to uterine contour.	Sum the 4 pocket depths = AFI	Record AFI and abnormalities: oligohydramnios AFI < 5 cm, polyhydramnios AFI > 24 cm
Middle Cerebral Artery (MCA) Doppler	Assess fetal cerebral blood flow for anemia/hypoxia	Supine	Grayscale to axial fetal head; identify circle of Willis and MCA. Activate color Doppler. Place PW Doppler gate over proximal third of MCA; ensure insonation angle <30°.	Capture PSV; compare with gestational-age reference ranges	Save waveform images; record PSV. Elevated >1.5 MoM suggests fetal anemia
Umbilical Artery (UA) Doppler	Evaluate placental resistance and fetal circulation	Supine	Grayscale to locate cord near fetal abdomen or placental insertion. Activate color Doppler. Place PW Doppler gate in a free-floating loop of UA.	Obtain waveforms and calculate S/D ratio, PI, RI; note absent/reversed end-diastolic flow	Save waveforms; document indices. Normal S/D ratio decreases with gestational age (e.g., <3.0 after 30 weeks)
Uterine Artery (UtA) Doppler - right & left	Assess maternal blood flow to placenta	Supine	Grayscale to locate external iliac artery in lower lateral uterus. Activate color Doppler to identify UtA crossing iliac vessels. Place PW Doppler gate in UtA.	Obtain waveforms; calculate PI; assess for early diastolic notch (abnormal if persistent after 24 weeks)	Record PI for both uterine arteries; normal PI < 1.45 after 24 weeks
Placental evaluation (Grayscale)	Assess placental location, structure, function	Supine	Grayscale scan of entire placenta. Note location (anterior/posterior/previa). Measure thickness (normal 2-4 cm). Check for calcifications, retroplacental clots, succenturiate lobes.	N/A	Document placental position, texture, thickness, and any pathology
Fetal Heart Rate (FHR)	Assess fetal cardiac activity	Supine	Grayscale to fetal heart (4-chamber view). M-mode: place M-line through ventricular wall. Spectral Doppler: place sample gate over mitral or aortic valve.	Calculate FHR using time between two consecutive beats	Record FHR (normal 120-160 bpm); note arrhythmias or decelerations

Study procedure

Participants with suspected pre-eclampsia (with enrollment blood pressure above 140/90 mmHg) will undergo further laboratory tests. A qualified laboratory technician shall collect 4 mL of venous blood for CBC, and a urine container shall be provided to the participant to collect a urine sample for urinalysis. Hemoglobin (Hb) level and platelet count will be recorded, while the presence of proteins in urine will be recorded after urinalysis. Participants with normal blood pressure at enrollment shall not undergo further laboratory tests unless requested by the attending physician. Data shall then be collected from the participants using the SonoAI application to record the necessary participant sociodemographic information, clinical, diagnosis, and laboratory data. The participant shall then proceed to have a Doppler USG examination. Data entered in the app shall be double-checked for completeness.

Image acquisition

Doppler USG images will be obtained using the Edan Acclarix LX25 Diagnostic Ultrasonography system, version 1.5, which is a high-resolution ultrasound machine with 2D/3D/4D and Doppler capabilities [19]. This system consists of a main unit and associated ultrasound transducers, including curvilinear, endovaginal, echo, and linear probes, that provide high-resolution images with internal storage capability. Ultrasonographic images generated from all patients examined are automatically stored and can be retrieved and converted to preferred shareable formats.

Doppler examination

**Figure 2:**
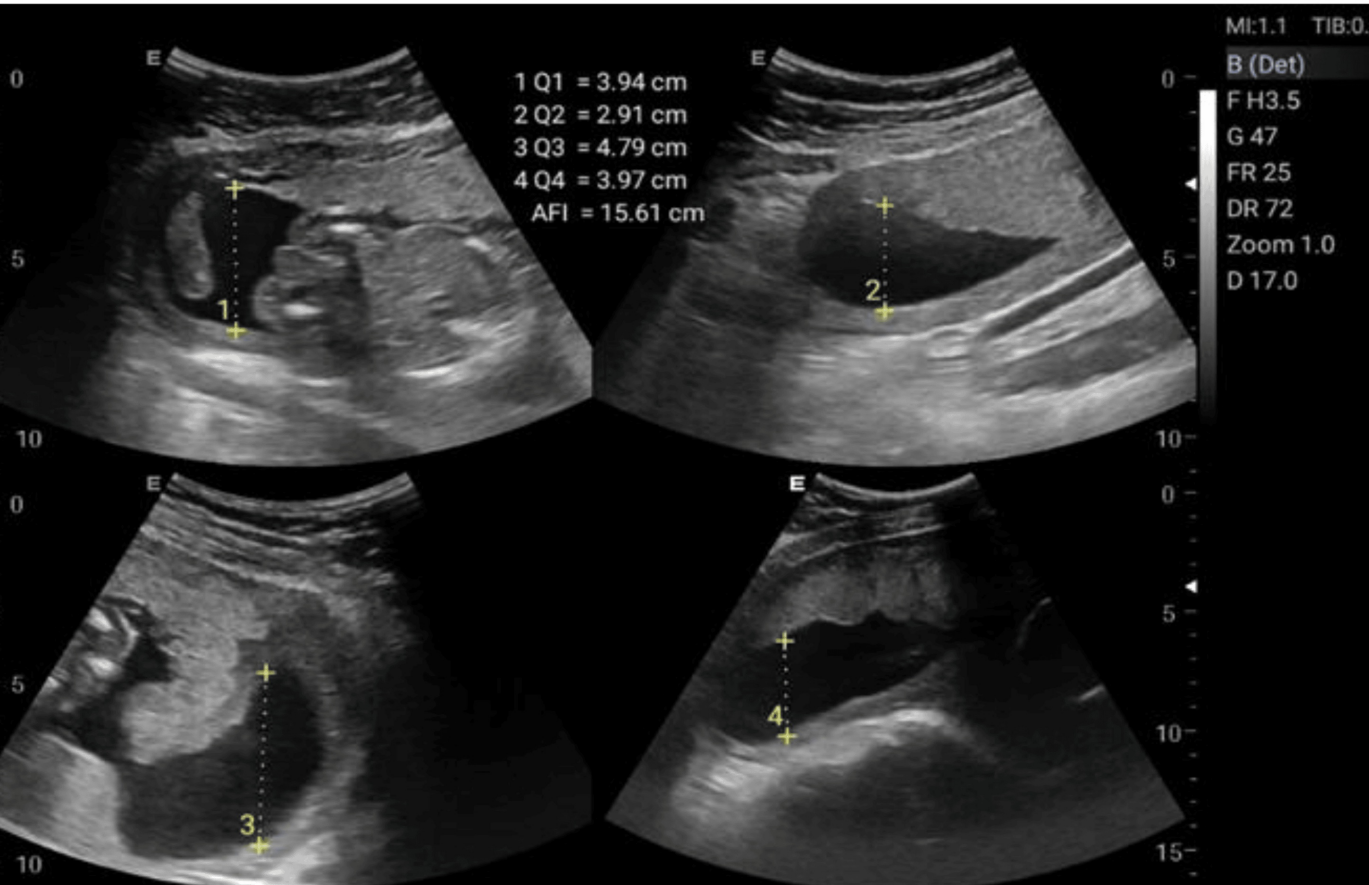
Amniotic fluid index (AFI) measurement.

**Figure 3:**
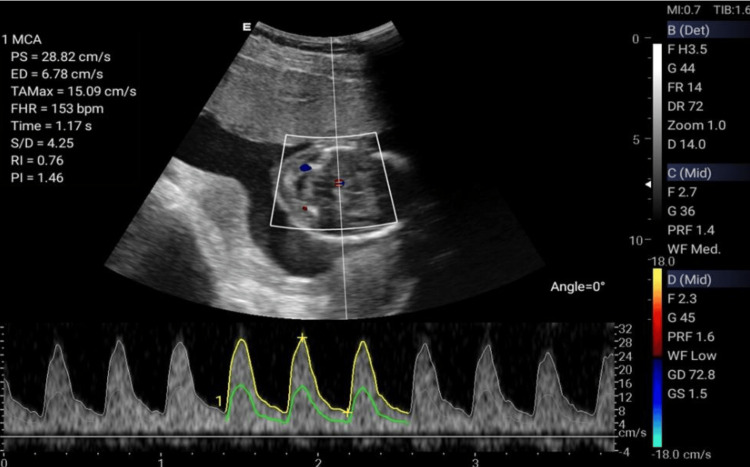
Middle cerebral artery (MCA) Doppler.

**Figure 4:**
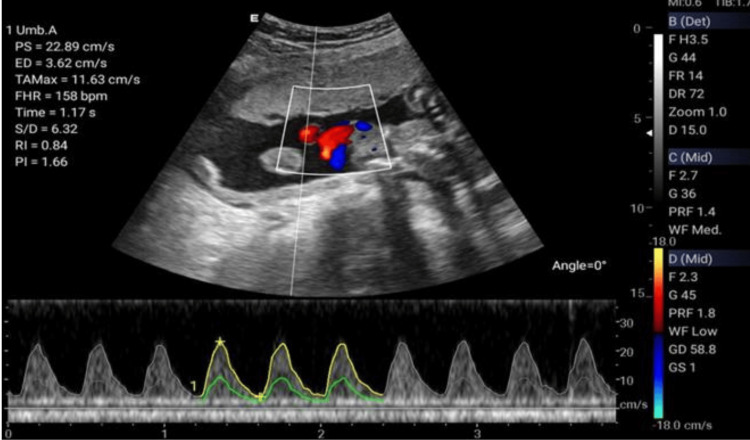
Umbilical artery Doppler.

**Figure 5:**
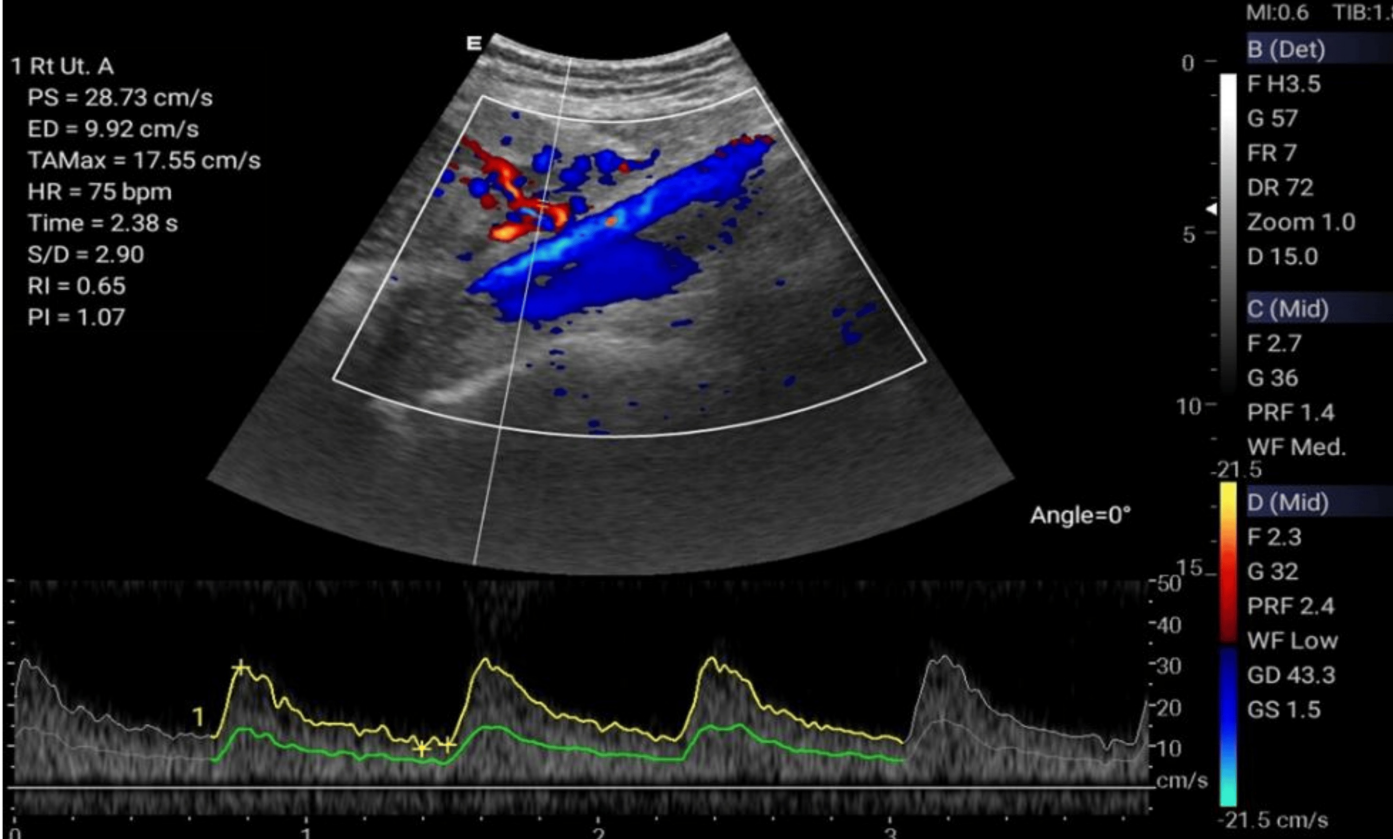
Right uterine artery Doppler.

**Figure 6:**
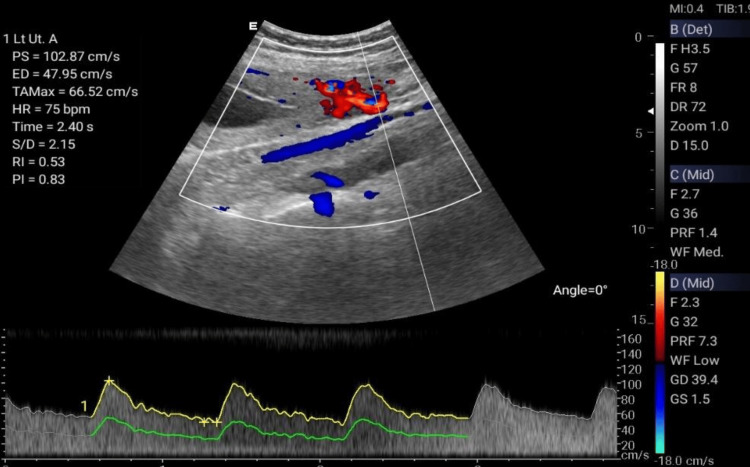
Left uterine artery Doppler.

**Figure 7:**
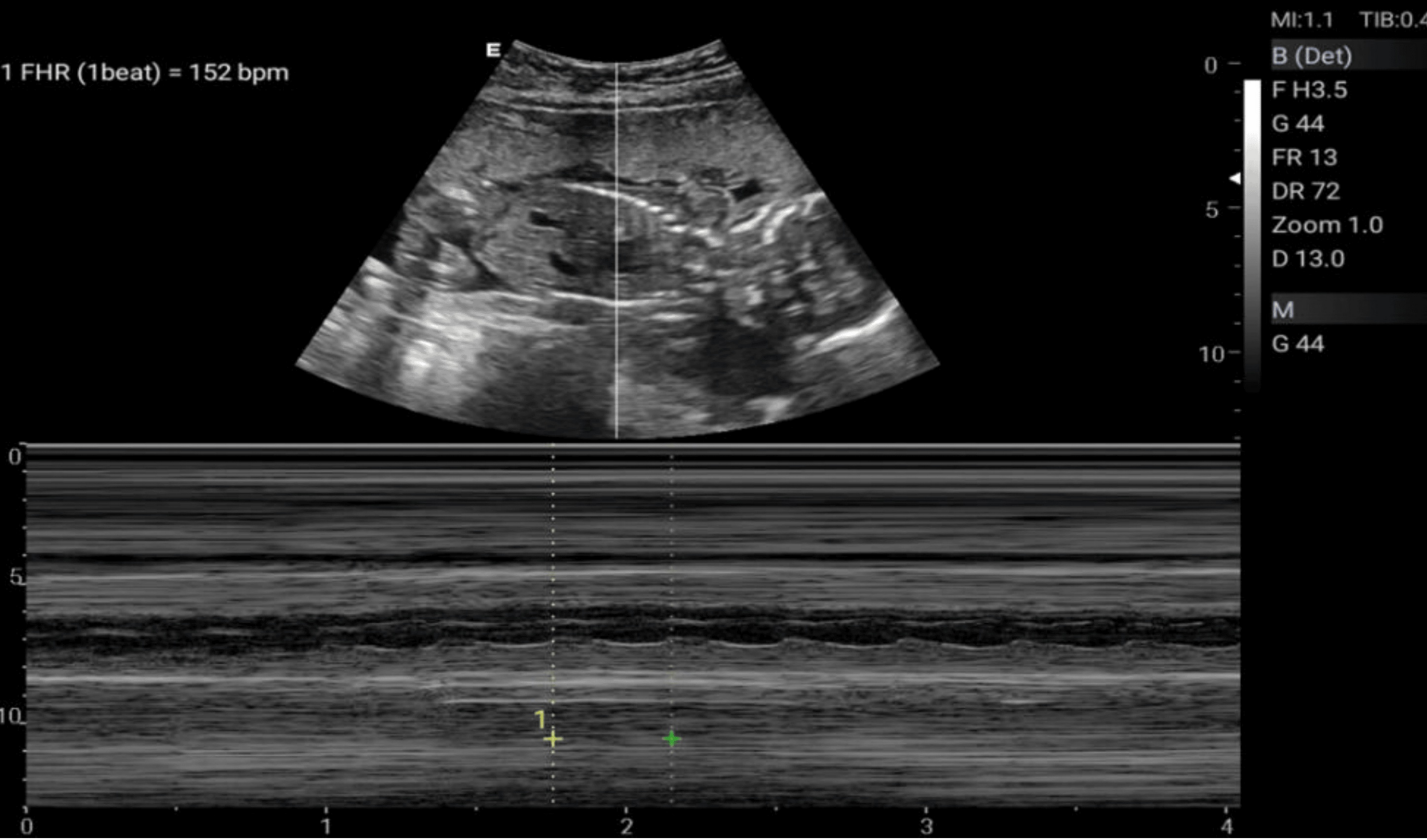
Fetal heart rate (FHR) measurement.

**Figure 8:**
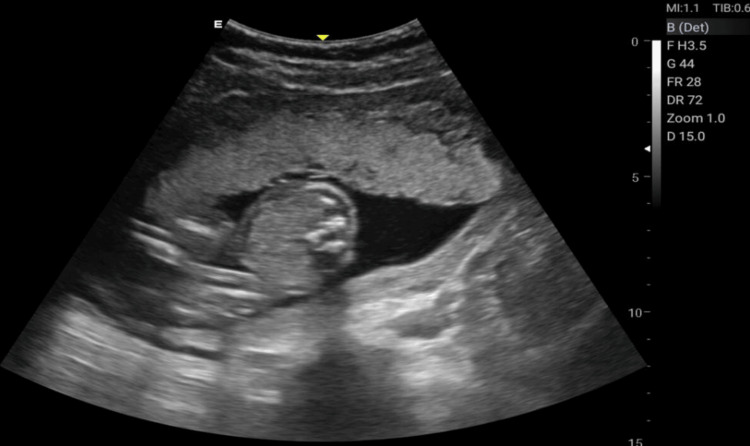
Placental ultrasound image.

The Doppler ultrasonographic examination shall be performed by qualified, well-trained radiographers using standard operating procedures and standardized protocols [20, 21] outlined in Table 1 to generate images relevant for clinical diagnosis. Ultrasonographic images for the amniotic fluid index (AFI) (Figure 2), middle cerebral artery (MCA) (Figure 3), blood flow characteristics in the umbilical artery (UA) (Figure 4), right uterine artery (Rt. Ut. A) (Figure 5), left uterine artery (Lt. Ut. A) (Figure 6), fetal heart rate (FHR) (Figure 7), and blood flow characteristics in the placenta (Figure 8) shall be obtained.

Image processing

Image processing to enhance quality will include elimination of extremely blurry and low-resolution images. Noise reduction will be done using adaptive filtering, which helps preserve edges and structural details, and wavelet transform, which decomposes the images into different frequency components, enabling noise to be isolated and removed without affecting the main image features. For artifact removal, segmentation techniques will be used to identify and isolate regions of interest to separate true anatomical structures from artifacts. For image enhancement, histogram equalization to improve image contrast and adaptive contrast enhancement, which modifies the brightness of different regions, will be used. Image normalization and standardization will involve pixel intensity normalization, which adjusts the range of pixel intensities; resolution standardization, which resamples all images to have a consistent resolution; and orientation correction, which ensures that all images have a consistent orientation [22].

Image labelling

The aim of this image labelling protocol is to provide standardized guidelines for labelling Doppler ultrasonographic images that shall be used to train an AI model for diagnosing pre-eclampsia and related complications. Expert radiologists, sonographers, and obstetricians will meticulously label key anatomical structures, fetal measurements, and potential abnormalities observed in the images related to pre-eclampsia. All images will undergo step-by-step image quality assessment, as shown in Table 2. Labellers shall ensure images are of high quality and in a standard image format, with standardized contrast and brightness for better AI training. We shall avoid unnecessary filtering to prevent alteration of radiological features. Images shall be converted to 16-bit grayscale, if necessary, for model consistency. We shall use images to develop a dataset with standardized labels to improve AI diagnostic accuracy.

**Table 2:** Steps in assessing image quality. Any image with a ‘No’ response to any criterion shall be excluded from the dataset.

Steps in Image Quality Assessment		
Does the image capture the correct anatomical structure or Doppler waveform intended for this study type?	Yes	No
Is the gestational age appropriate for interpreting the given image?	Yes	No
Is the image acquired in the correct standard plane/view?	Yes	No
Are there no significant artefacts that would affect interpretation of the image?	Yes	No
Is the image properly oriented?	Yes	No
Are the depth and zoom appropriate for visualizing the region of interest (ROI)?	Yes	No
Are image resolution, contrast, and brightness adequate for distinguishing key image features?	Yes	No
Are all key anatomical structures and diagnostic indicators, such as waveform peaks, fully visible?	Yes	No
Are all necessary image labels and annotations, including Doppler indices, visible and correct?	Yes	No
Is the image de-identified of any patient information?	Yes	No
Should this image therefore be included?	Yes	No

Labeling categories and definitions**

Each image shall be tagged with attributes, where applicable, as shown in Table 3. Each image shall be assigned a labeling category using relevant clinical parameters and Doppler indices shown in Table 4 below.

**Table 3:** Labeling categories and definitions. GA: Gestational age; AFI: Amniotic fluid index; MCA: Middle cerebral artery; RI: Resistance index; PI: Pulsatility index; CPR: Cerebroplacental ratio; FHR: Fetal heart rate; bpm: beats per minute; cm: centimeter(s).

Label name	Definition	Possible values
Gestational age (GA)	Weeks of pregnancy when the image was taken	Numeric (weeks)
Image type	Type of Doppler ultrasonographic scan	AFI; MCA Doppler; umbilical artery Doppler; right uterine artery Doppler; left uterine artery Doppler; placenta (grayscale); FHR
Resistance index (RI)	Blood flow resistance in an artery	Numeric (range)
Pulsatility index (PI)	A measure of flow resistance	Numeric
Amniotic fluid index (AFI)	Measurement of amniotic fluid volume	Numeric (cm)
Cerebroplacental ratio (CPR)	Ratio of MCA-PI to umbilical artery PI	Normal; Abnormal
FHR (bpm)	Fetal heart rate	Numeric

**Table 4:** Imaging labeling categories and Doppler indices.

Image Category	Description	Key Doppler Indices to Label	Normal Ranges	Label as:
Amniotic Fluid Index (AFI) Image	4-quadrant measurement of amniotic fluid volume	AFI (cm)	5-25 cm	<5: Oligohydramnios (suggestive of complications); >25: Polyhydramnios
Middle Cerebral Artery (MCA) Doppler	Measures fetal cerebral blood flow	Systolic/diastolic ratio (S/D)	4.4-6.0 (GA-dependent)	<4.0: Fetal hypoxia
		Resistance Index (RI)	0.70-0.85 (GA-dependent)	<0.70: Fetal hypoxia
		Pulsatility Index (PI)	1.5-2.0 (GA-dependent)	<1.0: Fetal hypoxia
		Cerebroplacental ratio (CPR)	>1.08 (may be GA-dependent by protocol)	<1.08: Fetal hypoxia
		MCA-PSV (cm/s)	(GA-dependent)	Low indicates brain hypoperfusion
Umbilical Artery Doppler	Assesses placental resistance	Systolic/diastolic ratio (S/D)	<3.0 (GA-dependent)	>3.0: Increased placental resistance
		Resistance Index (RI)	0.50-0.62 (GA-dependent)	>0.62: Risk of pre-eclampsia
		Pulsatility Index (PI)	1.0-1.6 (GA-dependent)	High: Risk of pre-eclampsia; Low: Fetal hypovolemia
Right Uterine Artery Doppler	Measures maternal-fetal blood flow on the right side	Systolic/diastolic ratio (S/D)	<2.6 (GA-dependent)	High: Risk of pre-eclampsia
		Resistance Index (RI)	<0.55 (GA-dependent)	>0.55: Impaired placental perfusion
		Pulsatility Index (PI)	<1.4 (GA-dependent)	>1.4: Fetal growth restriction
Left Uterine Artery Doppler	Measures maternal-fetal blood flow on the left side	Systolic/diastolic ratio (S/D)	<2.6 (GA-dependent)	High: Risk of pre-eclampsia
		Resistance Index (RI)	<0.55 (GA-dependent)	>0.55: Impaired placental perfusion
		Pulsatility Index (PI)	<1.4 (GA-dependent)	>1.4: Fetal growth restriction
Fetal Heart Rate (FHR) Image	Measures fetal heart rate	Heart rate (bpm)	110-160 bpm	<110: Bradycardia; >160: Tachycardia
Placental Image	Assesses placental maturity	Grading (1-3)	-	-
Worksheet	Summarizes indices of all images	Not applicable	N/A	N/A

Image annotation guidelines**

The aim of image annotation is to label visual data with tags, boundaries, or pixel-level marks for AI and machine learning models to understand, interpret, and learn from them. We shall use bounding boxes or segmentation masks to highlight key anatomical structures, apply standardized labels for each feature detected, and label regions of interest (ROI) for AI training.

Quality control and verification**

Each image shall mandatorily be reviewed by at least two medical experts before inclusion in the dataset. Labeling disagreements shall be resolved by a senior radiologist or obstetrician. AI-labeled images shall be compared against expert-labeled images for validation. Periodic inter-rater reliability (IRR) assessments should be conducted.

Data storage and management

All images shall be de-identified and anonymized to remove any remaining patient identifiers and assigned standard file names using a consistent format. All files shall be stored in formats such as DICOM, PNG, or TIFF (Figure 2-8) in a structured dataset for AI training.

Expected outcomes

We shall develop a high-quality labeled dataset to train an AI model for detecting preeclampsia-related complications. Improved accuracy and early detection of placental insufficiency, fetal distress, and maternal complications shall be enabled using this dataset. A scalable model that can be used in low-resource settings for real-time clinical decision-making shall be developed. Before starting the labelling process, a training session will be conducted for all labelers, including expert radiologists, sonographers, and obstetricians. Each labeled image will be reviewed by two experts to ensure accuracy and reduce subjective bias. In case of discrepancies, a third expert reviewer will be consulted. A comprehensive labeling guide will be provided, regular quality checks performed, and feedback collected from annotators.

Data management

De-identified relevant metadata will be summarized from the images and linked to the corresponding USG image. All images will be stored in the Digital Imaging and Communications in Medicine (DICOM) format and hosted by the DMAC at the Mbarara University Data Science Research Hub, on the Mbarara University of Science and Technology server.

Machine learning model development and evaluation

The machine learning model will be developed and evaluated through a five-stage pipeline comprising: (1) data acquisition and preprocessing, including image enhancement (noise reduction, contrast limited adaptive histogram equalization (CLAHE), resolution normalization, orientation correction), clinical metadata standardization, and data augmentation to improve diversity; (2) dataset splitting into training (70%), validation (15%), and test (15%) sets with stratification to preserve case proportions; (3) model architecture, exploring a custom convolutional neural network (CNN), transfer learning with residual network with 50 layers (ResNet50), DenseNet121, and EfficientNet-B3, and multimodal fusion of image and clinical data; (4) model training using Adam and stochastic gradient descent (SGD) optimizers, appropriate loss functions, early stopping, and learning rate scheduling; and (5) model evaluation with metrics such as accuracy, sensitivity, specificity, receiver operating characteristic (ROC) curve, AUC, and F1-score, statistical significance testing, and gradient-weighted class activation mapping (CAM) visualizations for explainability. Data governance will be overseen by a dedicated committee to ensure quality, mitigate bias, and maintain reproducibility.

Ethics and dissemination

Ethical clearance was obtained from the Research Ethics Committee (REC) at Mbarara University of Science and Technology (Registration number: MUST-2024-1321) and the National Council for Science and Technology in Uganda (Registration number: HS3890ES). Study site administrative clearance was obtained from the Hospital Director of Divine Mercy Hospital-Father Bash Foundation. All clinical metadata and images will be de-identified and anonymized before uploading to the DMAC.

## Results

The primary outcome of this research project will be the development of an annotated Doppler USG dataset. The study will generate a comprehensive, de-identified dataset of obstetric Doppler ultrasonographic images linked to structured clinical metadata. Images will include amniotic fluid index, fetal heart rate, umbilical artery, middle cerebral artery, uterine arteries, and placenta. Quality assurance will be achieved through preprocessing steps such as noise reduction, artifact removal, contrast enhancement, and normalization of resolution and orientation. Each image will undergo dual expert review, with discrepancies adjudicated by a third reviewer. Inter-annotator agreement will be quantified using reliability metrics (e.g., Cohen’s or Fleiss’ kappa), and annotation completeness rates will be reported. All images will be stored in DICOM format and archived securely on the DMAC infrastructure.

The secondary outcome will be a predictive AI tool for pre-eclampsia complications. This will include the development of a multimodal AI tool to predict maternal and fetal complications of pre-eclampsia, supporting early treatment and delivery decisions. Architectures explored will include a custom convolutional neural network and transfer learning models such as ResNet50, DenseNet121, and EfficientNet-B3. Fusion of image features with clinical metadata will be evaluated to enhance predictive performance. Training will employ stratified dataset splits (70% training, 15% validation, 15% testing), with augmentation strategies to improve diversity. Performance will be reported using accuracy, sensitivity, specificity, ROC-AUC, and F1-score, with statistical testing and confidence intervals. Explainability will be assessed through Grad-CAM visualizations, highlighting clinically relevant regions aligned with expert annotations.

## Discussion

This pilot study represents a novel, timely, and contextually relevant initiative to leverage emerging AI in the analysis and interpretation of ultrasonographic images for early detection and management of pre-eclampsia and related complications among pregnant women in Uganda. Maternal and perinatal outcomes remain suboptimal in Uganda [23, 24], partly due to delayed diagnosis and limited access to specialized imaging services [25, 26]. This study proposes to develop a rich dataset of USG images and introduce an innovative, context-adapted, AI-driven diagnostic and clinical decision-making support tool. This may ultimately improve maternal and neonatal outcomes.

Globally, AI has been successfully applied to obstetric USG in the determination of fetal sex, estimation of gestational age, and detection of congenital anomalies [27, 28]. This, however, has primarily occurred in high-income countries with advanced imaging technologies, strong digital infrastructure, and well-annotated datasets [29]. This study will create one of the first annotated datasets in sub-Saharan Africa, collected entirely from an African population. The dataset produced will fill a major gap in global AI research, which currently underrepresents low- and middle-income countries [30]. This will make the resulting AI model more relevant and adaptable to the clinical realities in Uganda and sub-Saharan Africa at large. This may also advance equity by ensuring that AI and machine learning models for obstetric care used in Africa are trained on African data.

The proposed study includes collecting images from pregnant women both with and without pre-eclampsia, which shall enable the creation of a comprehensive dataset and the development of a machine learning model capable of distinguishing between normal and pathological Doppler USG patterns [31]. The data collection process, involving structured image acquisition and annotation, shall ensure data quality and reproducibility, while the inclusion of relevant clinical data, such as blood pressure measurements, laboratory findings, and USS data, may add a critical layer of contextual information to support model training and validation.

From a health system perspective, this initiative aligns closely with the Uganda Ministry of Health Digital Health Strategy 2020/21-2024/25, which emphasizes innovations that enhance clinical decision-making, improve access to quality care, and strengthen digital health infrastructure [32]. AI is recognized as a transformative approach that is poised to improve the healthcare system, especially in areas with a scarcity of resources and healthcare workforce [33]. Furthermore, maternal health is a key pillar in national health plans and Sustainable Development Goal (SDG) targets for health [34]. The integration of AI into the maternal care pathway, particularly to aid the diagnosis of disorders of pregnancy such as pre-eclampsia, supports these broader goals. In settings where sonographers and radiologists are scarce, AI-driven interpretation of Doppler ultrasonographic images may improve diagnostic accuracy, optimize referral decisions, and enable timely interventions. These improvements are particularly crucial for pre-eclampsia, which remains a major contributor to maternal and perinatal morbidity and mortality in Uganda and across sub-Saharan Africa [35,36].

Despite the study’s strengths, several limitations must be acknowledged. As a pilot with a relatively small sample size of 150 participants yielding approximately 900 images, the generalizability of the resulting dataset and AI model may be limited. The model’s predictive performance will largely depend on the consistency of image quality and annotation, which may vary depending on operator skill and environmental factors. Furthermore, while the study will benefit from strong project infrastructure, routine adoption across Uganda’s health system would necessitate substantial investment in digital infrastructure, training, and regulatory oversight. Data privacy, informed consent, and clinical accountability for AI-generated recommendations are critical areas requiring formal policy guidance [37,38]. The Ministry of Health will need to develop and enforce standards for the validation, deployment, and monitoring of AI tools in clinical practice, including integration into existing health information systems.

This protocol presents a study that could provide valuable evidence for the application of AI in maternal healthcare within resource-limited settings. However, this pilot study will collect images using a cross-sectional design with a single interaction with enrolled participants, yet complications of pre-eclampsia develop over time, from 20 to 40 weeks of gestation. This study will provide a preliminary basis for longitudinal follow-up of patients with pre-eclampsia from diagnosis/onset until delivery, or until complications are detected, to support appropriate management.

## Conclusions

By developing and testing a locally trained machine learning model using Doppler USG images, this study proposes to address a critical diagnostic gap in obstetric care. The findings will not only inform future scale-up and integration of AI into Uganda’s maternal health services but also contribute to a growing body of evidence advocating for context-specific AI solutions to global health challenges. This work will demonstrate the feasibility of training a clinically relevant AI model in a low-resource setting using locally sourced and annotated Doppler ultrasound images. By integrating multimodal data (imaging plus clinical parameters), the model is expected to outperform image-only approaches in predictive accuracy.
